# Variability of the *mc1r* Gene in Melanic and Non-Melanic *Podarcis lilfordi* and *Podarcis pityusensis* from the Balearic Archipelago

**DOI:** 10.1371/journal.pone.0053088

**Published:** 2013-01-07

**Authors:** Joana M. Buades, Virginia Rodríguez, Bàrbara Terrasa, Valentin Pérez-Mellado, Richard P. Brown, Jose A. Castro, Antònia Picornell, M. M. Ramon

**Affiliations:** 1 Laboratori de Genètica, Universitat de les Illes Balears, Palma de Mallorca, Spain; 2 Departamento de Biología Animal, Universidad de Salamanca, Salamanca, Spain; 3 School of Natural Sciences and Psychology, Liverpool John Moores University, Liverpool, United Kingdom; George Washington University, United States of America

## Abstract

The association between polymorphism at the *mc1r* locus and colour variation was studied in two wall lizard species (*Podarcis lilfordi* and *P*. *pityusensis*) from the Balearic archipelago. *Podarcis lilfordi* comprises several deep mitochondrial lineages, the oldest of which originated in the Pliocene, while much shallower mitochondrial lineages are found in *P*. *pityusensis*. Here, we examined whether specific substitutions were associated with the melanic colouration found in islet populations of these species. Homologous nuclear sequences covering most of the *mc1r* gene were obtained from 73 individuals from melanic and non-melanic *Podarcis* from different populations (the entire gene was also sequenced in six selected individuals). MtDNA gene trees were also constructed and used as a framework to assess *mc1r* diversity. *Mc1r* showed greater polymorphism in *P. lilfordi* than in *P. pityusensis*. However, we observed no substitutions that were common to all melanic individuals across the two species. Only one significant association was detected in the *mc1r* partial sequence, but this was a synonymous A/G mutation with A alleles being more abundant in melanic populations. In addition, there were no associations between the main dominant phenotypes (green and brown, blue and yellow spots and ventral colour) and synonymous or non-synonymous substitutions in the *mc1r* gene. There was no statistical evidence of selection on *mc1r.* This study suggests no relationship between *mc1r* polymorphism and colour variation in Balearic *Podarcis.*

## Introduction

Although environmental stimuli can contribute to colour variation within species, most of this variation appears to be genetically controlled [1]. Molecular analyses are starting to reveal mutations associated with melanism in wild populations. In some birds and mammals, melanism seems to be associated with amino acid substitutions in the melanocortin-1 receptor (*mc1r*), a gene known to control the synthesis of melanin by melanocytes [2], [3]. The agouti-melanocortin 1 receptor pathway is a ligand receptor pair that modulates the amount and type of pigment produced by melanocytes (red/yellow pheomelanin or brown/black eumelanin). Genetic subtypes of *mcr* genes (of which *mc1r* is one) have high structural similarity. The majority of them seem to have originated early in vertebrate evolution before the divergence of ray-finned fishes and tetrapods [4]. The main structural properties of these genes have remained remarkably conserved over a period of at least 400 million years [4]. Gain-of function and/or deletion mutations in the *mc1r* locus are well recognized causes of melanism [5]. For example, a deletion in the *mc1r* gene explains melanism in squirrels [6]. In birds, Guo *et al.* [7] reported abundant polymorphism in the *mc1r* gene which was associated with black plumage in Hebei chickens. Different mutations in the *mc1r* gene also explain the brown phenotype in the cavefish, *Astyanax mexicanus* [8].

Several studies have recently addressed the *mc1r* gene and colour polymorphism in amphibians and reptiles. Three independent *mc1r* mutations (His^208^Tyr, Thr^170^Ile, and Val^168^Ile) are responsible for blanched coloration of three lizard species on the gypsum dunes of White Sands, New Mexico, where they are associated with melanin production in the species *Holbrookia maculata, Aspidoscelis inornata* and *Sceloporus undulatus* [9]. Although the same gene contributes to light phenotypes in these White Sands populations, the specific molecular mechanisms leading to reduced melanism production appear to be different. In contrast, sequence variation in *mc1r* does not explain melanism in the widespread amphibian *Rana temporaria* [10] nor does it appear to be involved in dorsal colour adaptations in two sympatric species of sand lizard (*Liolaemus*) that inhabit the south eastern coast of South America [11] or colour pattern in *Uta* lizards [12]. Some authors consider blue colouration to be a form of melanism in reptiles [13]. However the blue abdominal skin seen in several lizards is a sexually dimorphic trait that is more pronounced in males [14] and is attributed to eliciting a behavioural response in the observer [15]. Recently, an association between *mc1r* variants and brown scale colour phenotypes has been described in the European ocellated lizard, *Lacerta lepida* (*Timon lepidus*) [16].

The genus *Podarcis* is one of the most diverse and abundant reptile groups in southern Europe, with more than 20 currently recognized species [17], [18], and since the early works of Einer [19] has been known to contain several species that contain melanic populations. Melanic lizards and darker individuals in general, were originally thought to be associated with older island populations [19], [20], [21].

Two endemic species of *Podarcis* inhabit the Balearic Archipelago: *Podarcis lilfordi* in the Eastern Gymnesic Islands group (Mallorca, Menorca, Cabrera and their coastal islets) and *Podarcis pityusensis* in the Western Pityusic group (Ibiza, Formentera and coastal islets). Phylogenetic analyses have showed geographical structuring of mtDNA among insular populations of *P. lilfordi* with four main intraspecific lineages, the first of which diverged some 2.6 Ma [22], [23]. Divergence within *P. pityusensis* is more recent with the main Ibiza and Formentera clades sharing a common ancestor around 1 Ma ago [22], [23].

Eisentraut [21] described melanic populations of *P. lilfordi* from the islands of Aire (Menorca), and Foradada (Cabrera archipelago), as well as *P. pityusensis* from Bleda Plana [19]. He hypothesized that dark phenotypes were induced by a higher consumption of plant material. In contrast, Kramer [20] suggested an evolutionary explanation: dark colouration/melanism conveyed adaptive advantages through protection from harmful ultraviolet radiation, while enhancing heat absorption during cooler weather. Later, Hartmann [24] proposed that mutations that caused melanism originated before the coastal islets were separated from the main islands. The high degree of phenotypic variation (body size and colouration) among coastal islets is now well-established [25]. Several coastal islets host melanic populations of *P. lilfordi*, while a smaller number of islets also host melanic or very dark populations of *P. pityusensis*.

The purpose of the present paper was to investigate the putative association between polymorphism at the *mc1r* locus and colour phenotype in *Podarcis* from the Balearic Islands and therefore establish whether specific substitutions were associated with the melanic colouration of these populations.

## Materials and Methods

### Samples

A sample of 72 individuals from the genus *Podarcis* was analyzed (**Table 1**). These were: 1) 46 *Podarcis lilfordi* from 13 islands and islets corresponding to 7 light insular forms and 6 dark/melanic insular forms (from Mallorca, Menorca and Cabrera), 2) 22 *P. pityusensis* from 14 populations (from Ibiza and Formentera) with only two dark/melanic insular populations, 3) three *Podarcis tiliguerta* (from Corsica, France), and 4) one *P. filfolensis* (from Comino, Malta). The lizards from Corsica and Comino are light insular forms. A *Podarcis sicula mc1r* sequence was also available (Genbank: GU225767). Insular lizard populations were selected so that the most extreme phenotypes were included. All specimens were captured with official permits from national and regional organisms and the lizards released at the point of capture.

**Table 1:** Colour patterns of the different lizard populations.

	LOCALIZATION	POPULATIONS	DORSAL COLOUR						VENTRAL COLOUR				
			Brown	Green	Black	Dominant	Spots		Light	Dark	Blue	Orange	Blue Oce.
							Blue	Yellow					
***Podarcis lilford*** *i*	CABRERA ARCHIPELAGO	Cabrera	x	x		Brown			x		x		x
		Foradada			x	Black	x			x	x		
	MALLORCA	Dragonera	x	x		Brown			x			x	x
		El Toro	x			Brown			x				x
		Colomer			x	Black				x	x		
		Guardia			x	Black	x			x	x		
		Moltona			x	Black	x			x	x		
		Malgrats	x		x	Black				x	x		x
	MENORCA	Addaia	x	x		Green	x		x				x
		Binicodrell	x	x		Brown		x		x		x	x
		Rei	x	x		Brown		x	x				x
		Sanitja	x	x		Brown						x	
		Aire	x		x	Black	x			x	x		x
***Podarcis pityusensis***	IBIZA	Alga	x	x		Brown			x				x
		Bosc	x	x	x	Green	x			x		x	x
		Conillera	x	x		Green	x	x	x		x		x
		Dau Gran		x	x	Green	x	x		x	x		x
		Eivissa	x	x		Green			x			x	x
		Espartar	x	x	x	Green-Brown	x	x		x	x	x	x
		Espalmador	x	x		Green			x				x
		S. Josep	x	x	x	Green		x	x			x	x
		Bleda Plana			x	Black				x	x		
		Escull Vermell			x	Black	x			x	x		
	FORMENTERA	Cap Barberia	x	x	x	Green		x	x			x	x
		P.Trocadors	x	x		Brown			x			x	x
		Sa Pujada	x	x	x	Green		x	x			x	x
		St F. Xavier	x	x		Green	x	x	x		x		x
***Podarcis tiliguerta***		Foradada	x	x		Brown		x	x			x	
		Padodell	x	x		Brown		x	x			x	
		Stramari	x	x		Brown		x	x			x	
***Podarcis filfolensis***		Comino	x	x	x	Brown	x		x			x	x

Melanic populations are indicated by grey shading.

### Pigmentation Variation

Variation in pigmentation of the Balearic populations was classified from previous descriptions [25], [26]. Several colour characteristics were noted (within-islet variation was negligible for these characteristics). They included the presence of brown, black, green on the dorsum, dark/light ventral colouration and the presence of blue, yellow and orange spots (**Table 1**).

### DNA Extraction, Amplification and Sequencing

DNA was extracted during previous conservation genetics projects that described the mtDNA diversity within these lizards in order to underpin conservation strategies by the Balearic Islands Autonomous Government [25], [26]. A 720 bp fragment of *mc1r* gene was amplified with the forward primer MC1R-PF 5′-GGCNGCCATYGTCAANAACCGGAACC-3′ and the reverse primer MC1R-PR 5′-CTCCGRAAGGCRTAAATNATGGGGTCCAC-3′ (modified from Pinho *et al*., 2010 [27]). A second pair of primers was designed from the *Podarcis sicula* sequence to obtain the complete *mc1r* sequence (944 bp). The forward primer was 5′-ATGTCTGTGCCATCACCCCT-3′ and the reverse primer was 5′-GGTTCCGGTTCTTGACAATGGCNGCC-3′. The same PCR conditions were used for both sets of primers.

PCR reactions were performed in 25 µl volumes with 80 ng DNA, 1×PCR Buffer, 0.4 mM dNTPs, 0.3 µM of each primer and 0.5 units of DNA polymerase. PCR conditions were: 5 min at 92°C, 35 cycles of 30 s at 92°C, 30 s at 56°C, 90 s at 72°C; 5 min at 72°C. PCR products were purified using the Invitek MSB® Spin PCRapace (Invitek GMBH, Berlin, Germany). Both heavy and light strands were sequenced on an automated ABI 3130 sequencer using a Big Dye® v3.1 Cycle sequencing kit (Applied Biosystems, Foster City CA, USA). Sequence data have been deposited at the GenBank data library under accession numbers JX126622-JX126693.

The following partial mitochondrial genes were also amplified using PCR and sequenced: 12S rRNA, cytochrome *b* (two regions obtained separately), control region and an 800 bp (ND) fragment that included part of the ND1 gene, three tRNA genes, tRNA^Ile^, tRNA^Gln^_,_ and tRNA^Met^ and part of the ND2 gene. The total length of mitochondrial sequence analyzed for each animal was 2370 bp. We sequenced individuals from *P. pityusensis* (GenBank access EF694768, EF694794, EF694817, EF990552, EU006717, JX852045, JX852048, JX852051-JX852053, JX852055, JX852057-JX852059, JX852063, JX852066, JX852081, JX852091, JX852093-JX852094, JX852099, JX852104, JX852118, JX852120, JX852122-JX852123, JX852125, JX852127, JX852129), from *P. tiliguerta* (GenBank access JX852110-JX852111, JX852113-JX852114, JX852116-JX852117, JX852139-JX852141) and *P. filfolensis* (GenBank access JX852109, JX852112, JX852115, JX852138). Previously published *P. lilfordi* sequences [23] have also been used in this study.

Haplotype phases for *mc1r* were resolved for heterozygotic individuals using DnaSP software v5.10 [28] which implements an algorithm from the program PHASE [29], [30], [31]. The same software was used to obtain estimates of sequence diversity and compute the nucleotide diversity at synonymous, nonsynonymous, and silent sites, following Nei and Gojobori [32]. Neutrality was tested with Tajima’s D test [33] and Fu’s F test [34] using DnaSP [28].

A *mc1r* haplotype network was constructed using the program TCS v.1.21 [35] to examine whether or not melanism was associated with the overall *mc1r* genealogy. TCS creates a network using statistical parsimony [36], [37]. The probability of parsimony for linking haplotypes was set at the 95% level.

Phylogenetic trees were obtained using Bayesian inference on the haplotypes (MrBayes v.3.1.2 [38]). Two MCMC samplers were run in parallel (4 chains each, temperature (no lo llama el “heating parameter”) parameter set at 0.2) starting from a random tree for 1.3×10^6^ generations (samples recorded every 100 generations). In both sampling runs, stationarity of the Markov Chain was determined by stable split-standard deviations and stable sampled log likelihood values. The posterior sample of trees that followed burn-in were combined into a majority-rule consensus tree and used to estimate posterior node probabilities.

## Results

Assignment of the 27 Balearic populations and other *Podarcis* species to melanic and non-melanic sets is shown in **Table 1**. Individuals from Foradada, Guardia, Moltona, Aire and Escull Vermell islands within the melanic group of 8 populations also show blue spots. Melanic individuals from Aire and Malgrats populations also show brown dorsal spots and blue ventral ocelli. The non-melanic group comprises 19 populations. In this group, the most dominant dorsal colour is green (10 populations), followed by brown (8 populations). The presence of black, blue and/or yellow spots is less common. In general, non-melanic populations have light ventral colour.

**Figure 1:**
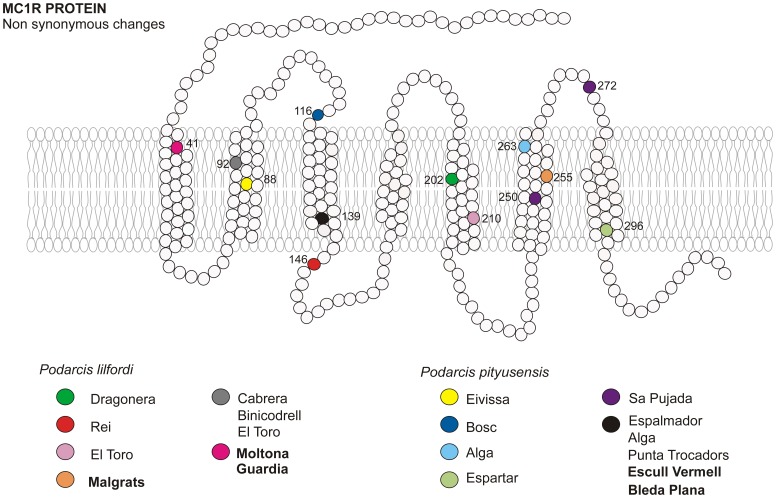
MC1R amino acid substitutions detected in *P. lilfordi* and *P. pityusensis*, and their locations relative to the cell membrane (modified from Garcia-Borron *et al*. [**43**] **).** Melanic populations are indicated in bold.

The 720 bp *mc1r* sequence provided 146 haplotypes, corresponding to 45 segregating sites, across *P. lilfordi*, *P. pityusensis*, *P. filfolensis* and *P. tiliguerta* specimens. The observed changes are displayed against the *P. sicula* reference sequence (**Table 2**). There were 32 synonymous and 13 nonsynonymous substitutions. We show the locations of nonsynonymous substitutions on the MC1R protein (**Fig. 1**). Substitutions which encode for different amino acids are V88I, V92E, V92L, D116N, I139V, F146L, M202V, Q210H, C250W, F255C, T263S, C272S, and A296P, mostly corresponding to the transmembrane domain of the protein.

**Table 2:** Synonymous and non synonymous (in bold) changes.

			Populations																												
			Cabrera	Dragonera	El Toro	Addaia	Binicodrell	Rei	Sanitja	Alga	Bosc	Conillera	Dau Gran	Eivissa	Espartar	Espalmador	S. Josep	C. Barberia	P. Trocadors	Sa Pujada	St F. Xavier	*P. tiliguerta*	*P. filfolensis*	Foradada	Colomer	Guardia	Moltona	Malgrats	Aire	Bleda Plana	E. Vermell
**Change positions (according to ** ***P. sicula*** **)**	**180**	**C**													G		G	G				G							G	G	G
	**210**	**G**				A																			A/G				A/G		
	**258**	**G**																						G/C							
	**261**	**C**		C/T		T																C/T							C/T		
	**262**	**G**												**A/G**																	
	**274**	**G**			**T**																										
	**275**	**T**	**A/T**				**C/T**																								
	**298**	**C**	C/T																					C/T							
	**318**	**T**	C	C	C			C/T	C	C	C	C	C	C	C	C	C	C	C	C	C	C		C	C	C	C	C		C	C
	**329**	**T**	**C**	**C**	**C**	**C**	**C**	**C**	**C**	**C**	**C**	**C**	**C**	**C**	**C**	**C**	**C**	**C**	**C**	**C**	**C**	**C**	**C**	**C**	**C**	**C**	**C**	**C**	**C**	**C**	**C**
	**333**	**C**	C/T																					C/T	C/T	T	T				
	**346**	**G**									**A/G**																				
	**351**	**C**								C/T						C/T	C/T			C/T	C/T							C/T	C/T		
	**375**	**C**	C/T	C/T																							C/T	C/T			
	**390**	**A**	A/G	A/G	G	G	A/G	G		A/G		G		A/G		A/G	G	A/G	A/G	G	G	G	G	G	A/G	G	A/G	A/G	G		
	**402**	**T**	C	C	C	C	C	C	C	C	C	C	C	C	C	C	C	C	C	C	C	C		C	C	C	C	C	C	C	C
	**405**	**T**	C	C	C	C	C	C	C	C	C	C	C	C	C	C	C	C	C	C	C	C		C	C	C	C	C	C	C	C
	**415**	**A**								**A/G**						**A/G**			**G**											**G**	**A/G**
	**438**	**C**						**G/C**																							
	**441**	**C**				T																T	C/T						C/T		
	**445**	**T**		C												C/T	C	C/T	C/T	C/T	C										
	**480**	**C**	T	C/T																			C/T								
	**483**	**C**																				T									
	**492**	**C**		C/T																											
	**540**	**C**	G/C	G/C																								G/C			
	**568**	**C**																					C/T								
	**594**	**C**																					C/T								
	**604**	**A**		**A/G**																											
	**629**	**G**			**G/C**																										
	**645**	**C**	C/T	C/T			C/T			C/T						C/T		C/T	C/T	T		C/T						C/T			
	**681**	**G**	A/G																			A		A/G	A/G	A	A				
	**684**	**G**					A/G	A/G																					A		
	**696**	**T**																				C/T									
	**726**	**C**					C/T																								
	**729**	**G**																						G/T							
	**735**	**C**					C/T																						T		
	**750**	**C**																		**G/C**											
	**764**	**T**																										**G/T**			
	**787**	**A**								**T**																					
	**795**	**G**	C/T	C	C	T	C	C	C	C	C	C	C	C	C	C	C	C	C	C	C	C	C	C	C	C	C	C	C/T	C	C
	**804**	**C**	C/T	C/T						C/T	T					C/T	C/T	C/T	C/T	C/T								C/T		T	C/T
	**814**	**T**																		**A/T**											
	**879**	**A**	C	C	C	C	C	C	C	C	C	C	C	C	C	C	C	C	C	C	C	C	C	C	C	C	C	C	C	C	C
	**885**	**C**												C/T		C/T			C/T					C/T		C/T	C/T				
	**886**	**G**													**C**																

Melanic populations are indicated by grey shading.

Two C-T transitions at sites 483 and 696 are specific to *P. tiliguerta*, and they represent synonymous substitutions: Val^161^ and Thr^232^. Two similar changes at sites 568 and 594 are present in *Podarcis filfolensis*. These changes correspond to Leu^190^ and Ile^198^.

Six sites (318, 329, 402, 405, 795 and 879) are divergent between *P. sicula* and *P. lilfordi*, *P. pityusensis*, *P. filfolensis* and *P. tiliguerta*. The T-C transition at site 329 is the only non-synonymous change. However, sites 318, 402 and 405 are not variable in *P. filfolensis*. Two populations of *P. lilfordi* (Addaia and Binicodrell) did not show substitutions at site 318. Thirty-nine sites within the *mc1r* gene fragment were variable within one (or more) of the insular populations of the Balearic Archipelago. Three sites in the *mc1r* sequence could be considered potential mutational hot spots (SNPs): c.390A>R, c.645C>Y and c.804C>Y positions.

We tested for associations between *mc1r* variants and colour categories. Because of the high mc1r diversity observed, PHASE could not assign haplotype identities to all genotypes with high probabilities. Tests were therefore based on direct sequence, incorporating heterozygotic sites, rather than on phased haplotypes. First, we examined whether any specific changes in the sequence were associated with melanic phenotypes. No substitutions were common to all melanic individuals. A contingency table test was used to analyze possible associations between each polymorphic position and the melanic phenotype. The only significant deviation association was detected in the 681nt (χ^2^ = 25.17, P<0.001) but this was an A/G synonymous change. Alleles containing an A were more abundant in melanic populations.

**Figure 2:**
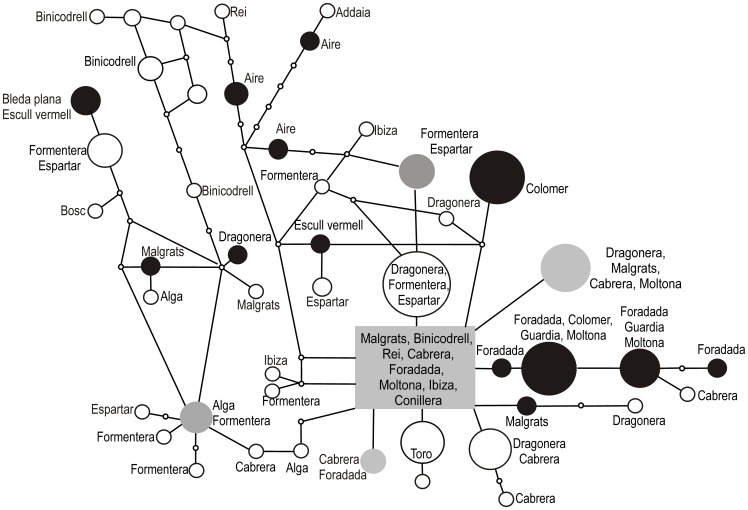
Network showing relationships among *mc1r* haplotypes observed in Balearic populations. Alleles from melanic populations are represented as black circles, and non-melanic populations as white circles. Alleles that were found in both melanic and nonmelanic populations are represented as grey circles.

The DNA network results are shown in Figure 2. The melanic populations of Foradada, Colomer, Guardia and Moltona (*P. lilfordi*) share some common synonymous changes at Thr^111^ and Ser^227^. However, these substitutions are not shared with the *P. lilfordi* from Aire and Malgrats, or Escull Vermell and Bleda Plana melanic populations of *P. pityusensis*.

We also examined whether substitutions in the first 210 bp of the gene might be associated with melanism by sequencing the entire *mc1r* gene for a smaller group of 6 individuals from 3 melanic (Moltona, Guardia and Malgrats) and 3 non-melanic (Cabrera, Dragonera and Formentera (P. Trocadors)) islets. Sequences were compared to *P. sicula* sequence and seven substitutions were detected. Six of these substitutions were present in all populations and were synonymous: Pro^14^, Pro^21^, Asn^24^, Leu^43^, Phe^52^ and Lys^64^. One non-synonymous change, G41D, was detected (**Fig. 1**). However, this caused an amino acid substitution that had no effect on protein charge. This substitution was observed in the melanic populations from Moltona and Guardia but not in the melanic population from Malgrats. There were no indels within the initial 210 base pairs of the *mc1r* sequence. There were no associations between phenotype categories (green and brown, blue and yellow spots and ventral colour) and the observed amino acid or synonymous substitutions in *mc1r*.

### Gene Diversity and Selective Effects

**Figure 3:**
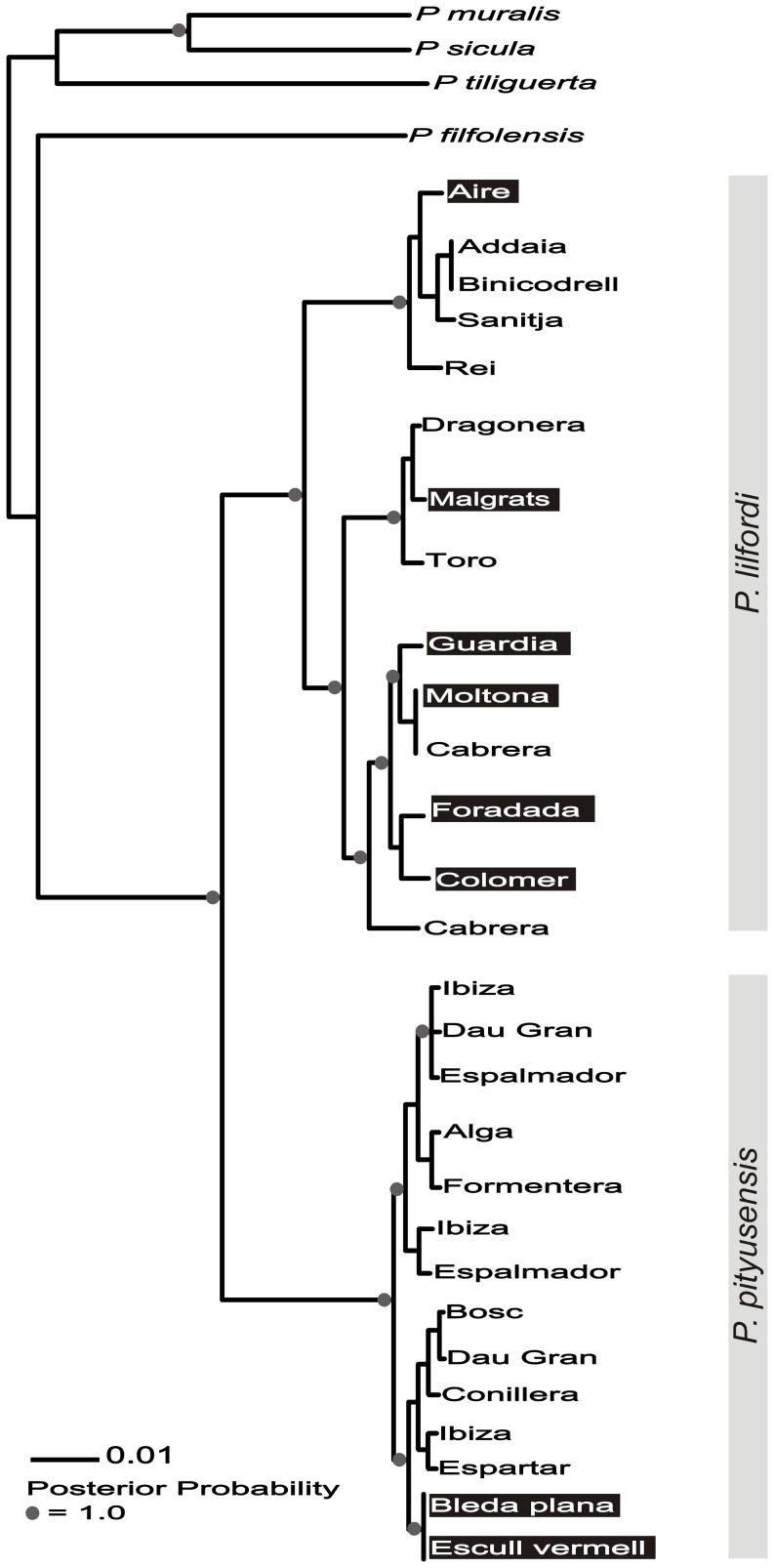
The mtDNA consensus tree inferred by Bayesian inference. Melanic *P. lilfordi/P. pityusensis* populations are highlighted in black. Circles are placed on nodes in which posterior probabilities were ≥0.9.

The mitochondrial gene tree that was inferred for the same individuals is shown in **Figure 3**. The tree is concordant with the two species and also shows clear island phylogeographical patterns within species. Both melanic and non-melanic populations were found in the three main mtDNA lineages in *P. lilfordi* but were only detected within a single mtDNA lineage in *P. pityusensis*. The *mc1r* gene tree shows low posterior support for some nodes. However, splits in the tree do not correspond to melanic/non-melanic population divisions. Unlike the mtDNA tree, the major split within the tree does not correspond to the two species.

Table 3 shows the diversity estimates for the *mc1r* gene and the mtDNA. Both loci have greater diversity in *P. lilfordi* than in *P. pityusensis*. Also, the melanic insular populations are less diverse than the non-melanic ones.

**Table 3:** Genetic diversity parameters based on *mc1r* gene sequences (720 bp) and mtDNA (2370 bp).

	MC1R								mtDNA							
	N	S	h	Hd	K	Pi	D	F	N	S	h	Hd	K	Pi	D	F
***P. lilfordi***	92	30	35	0.955 (0.009)	3.634	0.005 (0.001)	−1.242^ns^	−1.237^ns^	36	154	23	0.965 (0.016)	47.535	0.020 (0.001)	0.929^ns^	1.271^ns^
***P. pityusensis***	44	14	23	0.958(0.014)	3.050	0.004(0.001)	−0.163^ns^	−0.324^ns^	21	45	17	0.976(0.023)	9.533	0.004(0.001)	−0.943^ns^	− 1.386^ns^
Other species	8	12	6	0.929(0.084)	4.464	0.006(0.001)	−0.178^ns^	0.025^ns^	4	259	3	0.833(0.222)	136.500	0.058 (0.023)	−0.537^ns^	− 0.595^ns^
Melanic	44	22	18	0.900(0.031)	3.476	0.005(0.001)	−1.029	−1.143^ns^	18	202	10	0.935(0.032)	56.451	0.024 (0.005)	−0.244^ns^	1.143^ns^
Non melanic	92	34	43	0.968(0.007)	3.582	0.005(0.001)	−1.493^ns^	−1.972*	39	226	30	0.977(0.014)	78.260	0.033(0.002)	1.591^ns^	1.424^ns^

N = number of sequences: S = number of segregating sites; h = number of haplotypes; Hd = haplotype diversity; K = number of pairwise differences; Pi = nucleotide diversity; D Tajima’s D (1989) Fu’s F and Li’s F (1993). SE is indicated in parentheses.

ns not significant, * P<0.05.

The melanic individuals belong to *P. lilfordi* and *P. pityusensis* insular populations.

No statistically significant signature of selection was detected using Tajima’s D test [33]. Values were slightly negative, with the exception of *P. lilfordi* and non-melanic groups that present slightly positive D values for mtDNA. The results from Fu’s and Li’s tests [34] were similar, except in the case of the non-melanic group (for the *mc1r* gene), for which F statistic is negative, and significant at 5% (P<0.05).

## Discussion

Substitutions in the *mc1r* gene of endangered Balearic Island *Podarcis* lizards do not appear to be related to either melanism or other components of the considerable colour pattern variation among islands.

Recent studies on pigmentation genes and their functions have provided evidence that pigment gene function is largely conserved across vertebrate taxa and can influence adaptive coloration, often in predictable ways [1]. The *mc1r* gene is highly conserved among vertebrates and has a relatively simple genetic structure. This has facilitated its identification in a diversity of taxa, including lizards. The majority of these studies try to associate a punctual non-synonymous sequence change with a discrete colour polymorphism.In some cases, identical mutations at homologous positions in diverse taxa have been found to led to the same or similar phenotypes [1].

The *mc1r* gene is polymorphic in the studied populations. We found 45 variable positions with respect to the published *mc1r* gene sequence of *P. sicula* and three of these positions could be considered as hot spots due to their high mutation frequency. As expected under neutral evolution, synonymous changes are most numerous but thirteen substitutions encode for different amino acids, and most of them correspond to the transmembrane domain of the protein. Much of the genetic diversity in *mc1r* appears to reflect the patterns observed in the mtDNA, which have been interpreted in terms of the historical biogeography of these species [22], [23]. For example, *P. lilfordi* shows much greater genetic diversity (in both loci) than *P. pityusensis*. Previous mtDNA analyses showed that was likely to have originated from ancient isolation on the major islands of Mallorca, Menorca and Cabrera during the Pliocene [22], [23].

Nunes *et al.* [16] detected two associations between *mc1r* variants and ecologically relevant phenotypes in the European ocellated lizard *Lacerta lepida*, a genus related to *Podarcis.* The first is a non-conserved and derived substitution (T162I) associated with the presence of brown scales (“*nevadensis*” phenotype), while the second substitution (S172C) was associated with the presence of black scales in both *L. l. lepida* and *L. l. iberica*. However, they did not detect mutations associated with the higher proportion of black scales in *L. l. iberica*. Here, the nucleotide positions 162 and 172 were not variable among the very polymorphic populations of *Podarcis*.

With some exceptions [5] melanism is also associated with substitutions at the *mc1r* locus in a variety of mammals and birds, including domestic [39], [40] and wild species [41]. In this case structural mutations (deletions) are thought to be responsible for the melanic phenotype. There were no deletions in the *mc1r* gene sequence in melanic populations of *Podarcis lilfordi* and *Podarcis pityusensis*, suggesting that this is not the case here. The unique substitutions that we have observed in the melanic populations: Foradada, Colomer, Guardia and Moltona (*P. lilfordi*), are synonymous changes at Thr^111^ and Ser^227^. However, the *P. lilfordi* Aire and Malgrats island populations, and the *P. pityusensis* Escull Vermell and Bleda Plana island populations, are melanic, but do not share these substitutions. It is therefore very difficult to believe they play a role in melanism in any of these species. Similar findings have recently been reported for the side-blotched lizard, *Uta stansburiana* [12].

The presence of a dark phenotype is thought to be a relict character in cordylic lizards [42]. However, it has been hypothesized that this character is under quite strong selection in *Podarcis* due to its impact on thermoregulation [20]. Given the low prevalence of melanism in other *Podarcis*, it seems unlikely that melanism is the ancestral condition for these species. If we assume that the mtDNA branching pattern reflects the true species/population history then the most parsimonious explanation is that the ancestral condition is the non-melanic colour seen in most other *Podarcis*. If this is the case here then melanism has clearly evolved several times within Balearic *Podarcis*. However, our statistical tests on mc1r provided no support for the hypothesis that that this can be attributed to different selection regimes on different melanic populations.

*Podarcis* coloration therefore seems to be attributable to other loci. For example, agouti signaling protein (*asip*) is important in melanin synthesis and multiple mutations in this gene are associated with colour variation. However, compared with *mc1r*, far fewer studies have been carried out on wild populations and also the molecular changes associated with colour variation are different with both coding and regulatory regions being implicated. To date, agouti-like sequences have not been reported in reptiles. In addition to melanin pigments, animal coloration can involve carotenoid pigments and pterins, but the genetic mechanisms involved in these pathways are poorly understood [1].

Balearic Island *Podarcis* populations exhibit a wide variety of colour variants, with morphs ranging from completely melanic to quite light-coloured individuals, although green-brown pigmentation is the most frequent morph. Sequencing of the *mc1r* gene in individuals with different morphological phenotypes has not revealed a clear correlation between mutations and/or deletions and these different colour morphs.
