# Correction: Variability of the *mc1r* Gene in Melanic and Non-Melanic *Podarcis lilfordi* and *Podarcis pityusensis* from the Balearic Archipelago

**DOI:** 10.1371/annotation/203b494a-8670-4b4a-8f4f-a101cf03dcc8

**Published:** 2013-09-13

**Authors:** Joana M. Buades, Virginia Rodríguez, Bàrbara Terrasa, Valentin Pérez-Mellado, Richard P. Brown, Jose A. Castro, Antònia Picornell, M. M. Ramon

The phrase "Melanic populations are indicated by grey shading" should be ignored as the shading for the tables no longer corresponds to melanic populations. 

